# Reliable detection of subendocardial ischemia by high-resolution end-systolic first-pass perfusion imaging in the absence of obstructive coronary disease

**DOI:** 10.1186/1532-429X-18-S1-O12

**Published:** 2016-01-27

**Authors:** Behzad Sharif, Rohan Dharmakumar, Daniel S Berman, Debiao Li, C Noel Bairey Merz

**Affiliations:** 1 Biomedical Imaging Research Institute, Cedars-Sinai Medical Center, Los Angeles, CA USA; 2 Cedars-Sinai Heart Institute, Los Angeles, CA USA

## Background

Among the patients without known disease undergoing invasive angiography, nearly 40% do not have coronary artery disease (CAD) [1]. A possible underlying etiology for such patients is ischemic heart disease associated with coronary microvascular dysfunction (CMD). Despite intense interest and recent advancements, reliable diagnosis of CMD on the basis of first-pass perfusion (FPP) cardiac MRI is an ongoing challenge. We hypothesized that high-resolution systolic FPP imaging can detect diffuse vasodilator-induced subendocardial defects and transmural perfusion gradients (TPGs) consistent with CMD in a swine model of diet-induced diabetes with no obstructive CAD. To this end, we optimized and tested a recently introduced high-resolution FPP method capable of imaging all myocardial slices at the end-systolic phase.

## Methods

Yucatan mini-pigs (n = 8 males) were fed either a high-fat high-sugar diet (n = 4 "HFHS" group) or a normal chow diet (n = 4 "control" group) for 20 weeks. Compared to controls, the HFHS pigs were obese (68 ± 8 kg vs 45 ± 7), and had abnormally elevated fasting glucose (177 ± 19 mg/dL vs 94 ± 12) and insulin levels, indicating early-stage type-2 diabetes and expected to have CMD based on previous studies [2]. Obstructive CAD was ruled out in all pigs using invasive angiography on the day of the MRI study, consistent with their normal serum lipid levels. There was no difference between baseline arterial systolic blood pressure between the two groups, suggesting absence of hypertension in the HFHS group. Adenosine stress/rest FPP data was acquired using a recently introduced T1-weighted steady-state ungated FLASH sequence [3] with optimized slice-interleaved radial sampling (acquired in-plane resolution: 1.2 × 1.2 mm^2^). Retrospective end-systolic self-gating was performed automatically (temporal resolution: 32 ms) and compressed sensing combined with apodization was used to generate dark-rim-minimized images. TPG analysis was performed according to the established approach [4].

## Results

**Figure 1:**
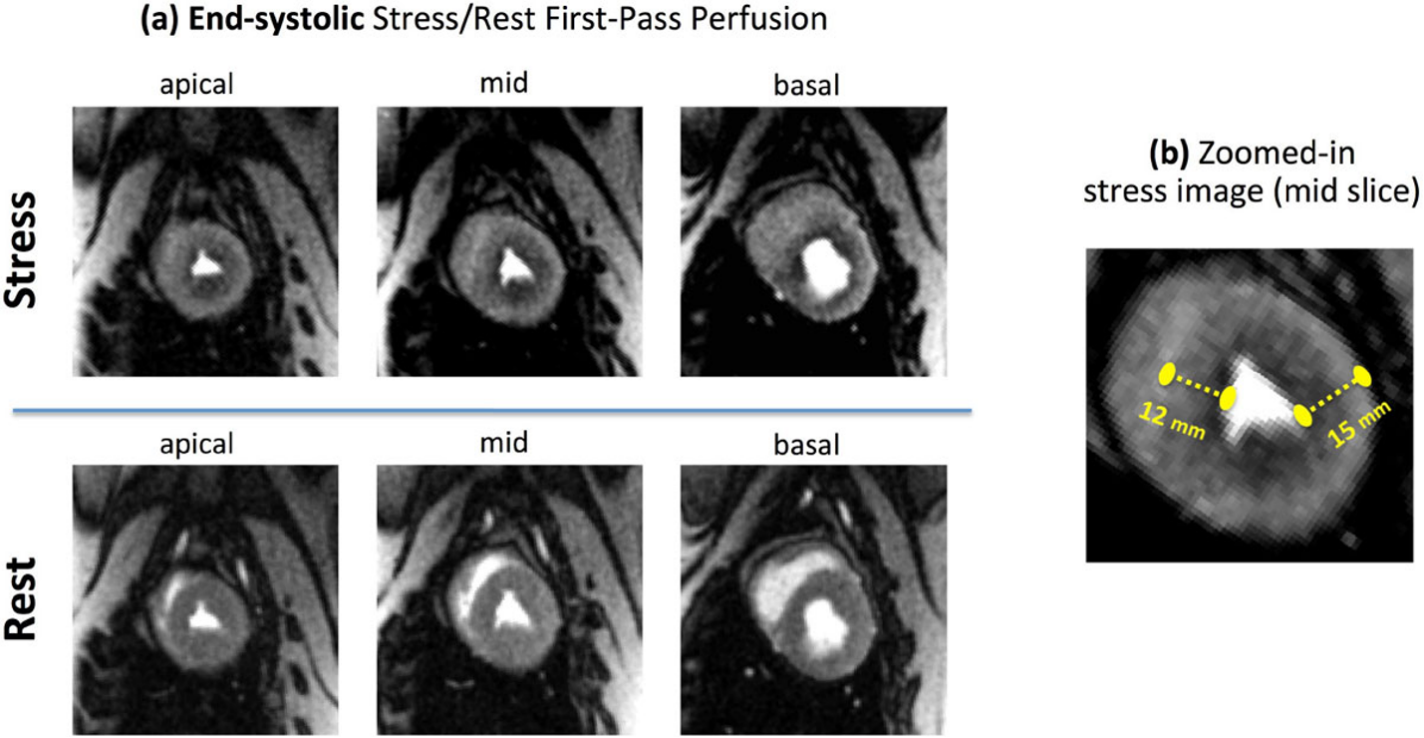
**Representative adenosine stress/rest myocardial perfusion images in a HFHS pig without obstructive CAD and expected to have coronary microvascular dysfunction (CMD), demonstrating the ability to visually detect diffuse vasodilator-induced hypoperfusion at the subendocardial layer**. **(a,b):** End-systolic adenosine stress/rest first-pass perfusion images with a prescribed 1.2 × 1.2 mm in-plane resolution corresponding to the peak myocardial-enhancement phase (no image interpolation used). The self-gated steady-state imaging approach enables reconstruction of all myocardial slices at the end-systolic phase. Stress-induced subendocardial perfusion defects are diffusely present in all myocardial slices and vessel territories, consistent with CMD. **(c):** Zoomed-in stress image (mid slice) shows the highly-resolved myocardium with multiple "defect pixels" present across the subendocardium: the measured endocardial-epicardial distance is nearly 10 times the size of a single image pixel.

**Figure 2:**
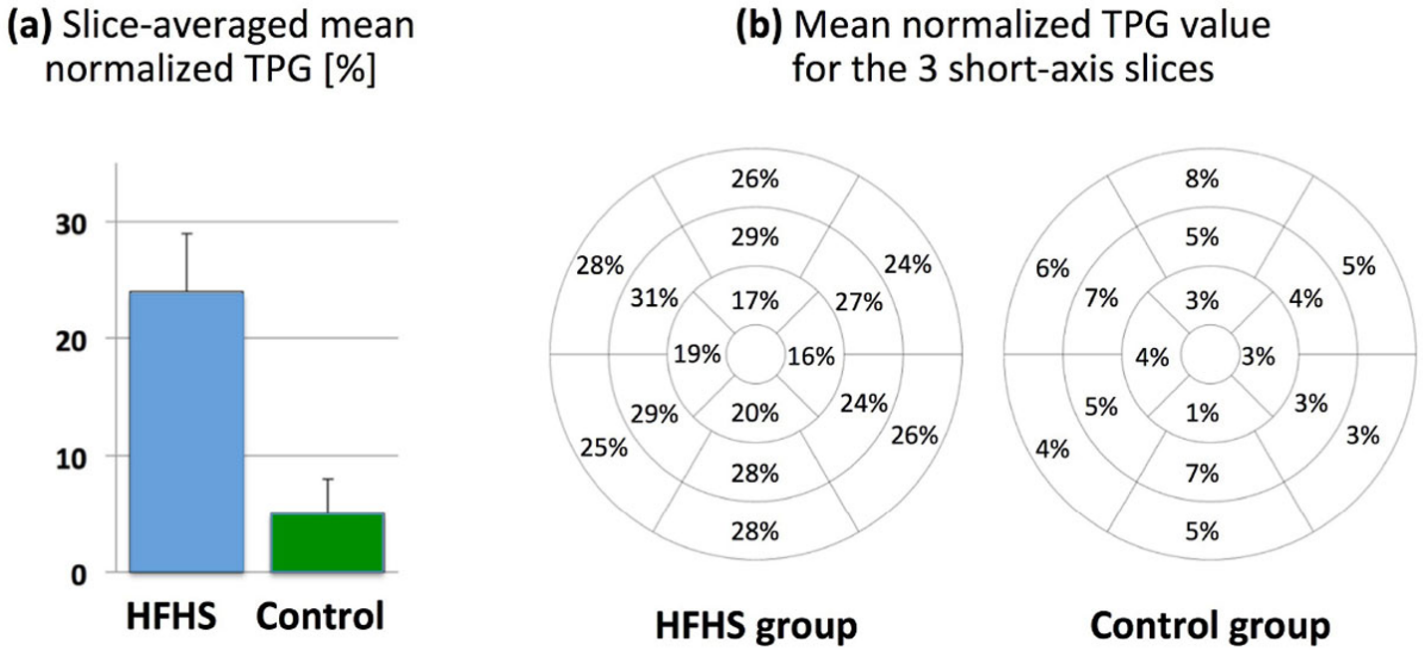
**Results for quantitative assessment of myocardial perfusion gradients using the previously established transmural perfusion gradient (TPG) analysis method**. **(a):** Comparison of slice-averaged mean TPG (normalized value in percentage) for the HFHS versus control pigs, showing a significantly higher TPG value for the HFHS group in comparison to controls (24% ± 5% vs 5% ± 3%). **(b):** Bull's eye map of mean TPG (normalized value for each of the 16 myocardial segments) averaged across the n = 4 pigs in each group. The TPG analysis results are consistent with the visual assessment (example shown in Fig. 1) and demonstrate a marked stress-induced transmural gradient in the HFHS pigs compared with controls.

Representative myocardial perfusion images for a HFHS pig are shown in Fig 1 demonstrating presence of global adenosine-induced subendocardial perfusion defects (see caption). Visual assessment of stress FPP images (2 blinded readers in consensus) showed a delayed wash-in of contrast in the subendocardium vs. subepicardium for all HFHS pigs and normal perfusion in the control pigs. TPG analysis (Fig 2) demonstrated a significantly higher mean TPG across all myocardial segments in HFHS pigs compared to controls (24% ± 5% vs 5% ± 3%).

## Conclusions

We demonstrated that subendocardial ischemia and stress-induced TPGs can be visually detected in the absence of obstructive CAD using an optimized FPP approach. The combination of high isotropic in-plane resolution, end-systolic imaging of all slices, and dark-rim-minimized reconstruction enables reliable detection of subendocardial defects. Our results suggest that this methodology may be a promising approach for accurate diagnosis of CMD in clinical settings.

